# Biallelic *ATP2B1* variants as a likely cause of a novel neurodevelopmental malformation syndrome with primary hypoparathyroidism

**DOI:** 10.1038/s41431-023-01484-9

**Published:** 2023-11-06

**Authors:** Patrick Yap, Lisa G. Riley, Purvi M. Kakadia, Stefan K. Bohlander, Ben Curran, Meer Jacob Rahimi, Salam Alburaiky, Ian Hayes, Henry Oppermann, Cristin Print, Sandra T. Cooper, Polona Le Quesne Stabej

**Affiliations:** 1 Department of Molecular Medicine and Pathology, University of Auckland, Auckland, New Zealand; 2 Genetic Health Service New Zealand - Northern hub, Auckland, New Zealand; 3 Rare Diseases Functional Genomics, Kids Research, The Children’s Hospital at Westmead and The Children’s Medical Research Institute, Sydney, NSW 2145 Australia; 4 Specialty of Child & Adolescent Health, Sydney Medical School, University of Sydney, Sydney, NSW 2006 Australia; 5 Leukaemia and Blood Cancer Research Unit, Department of Molecular Medicine and Pathology, University of Auckland, Auckland, New Zealand; 6 Institute of Human Genetics, University of Leipzig Hospitals and Clinics, Leipzig, 04103 Germany; 7 Kids Neuroscience Centre, Kids Research, Children’s Hospital at Westmead, Sydney, NSW 2145 Australia; 8 The Children’s Medical Research Institute, 214 Hawkesbury Road, Westmead, NSW 2145 Australia

**Keywords:** Disease genetics, Medical genomics

## Abstract

*ATP2B1* encodes plasma membrane calcium-transporting-ATPase1 and plays an essential role in maintaining intracellular calcium homeostasis that regulates diverse signaling pathways. Heterozygous de novo missense and truncating *ATP2B1* variants are associated with a neurodevelopmental phenotype of variable expressivity. We describe a proband with distinctive craniofacial gestalt, Pierre-Robin sequence, neurodevelopmental and growth deficit, periventricular heterotopia, brachymesophalangy, cutaneous syndactyly, and persistent hypocalcemia from primary hypoparathyroidism. Proband-parent trio exome sequencing identified compound heterozygous *ATP2B1* variants: a maternally inherited splice-site (c.3060+2 T > G) and paternally inherited missense c.2938 G > T; p.(Val980Leu). Reverse-transcription-PCR on the proband’s fibroblast-derived mRNA showed aberrantly spliced *ATP2B1* transcripts targeted for nonsense-mediated decay. All correctly-spliced *ATP2B1* mRNA encoding p.(Val980Leu) functionally causes decreased cellular Ca^2+^ extrusion. Immunoblotting showed reduced fibroblast ATP2B1. We conclude that biallelic *ATP2B1* variants are the likely cause of the proband’s phenotype, strengthening the association of *ATP2B1* as a neurodevelopmental gene and expanding the phenotypic characterization of a biallelic loss-of-function genotype.

## Introduction

*ATP2B1* encodes the plasma membrane calcium-transporting ATPase 1 (ATP2B1 or PMCA1), which regulates intracellular Ca^2+^ concentration by Ca^2+^ extrusion. Mammals express four Plasma Membrane Calcium ATPases (ATP2B1-4) encoded by four genes (*ATP2B1-4*). Targeted deletions of *Atp2b1-4* in mice show distinct phenotypes, highlighting the functional specificity of each gene [1, 2]. *ATP2B1* is expressed ubiquitously and is the earliest isoform expressed in embryonic development [3]. Homozygous *Atp2b1* knockout (ko) mouse is embryonically lethal before organogenesis [4, 5], whereas heterozygous *Atp2b1-ko* mice have hypertension, hypocalcemia with decreased intact parathyroid hormone (PTH) levels, and increased bone mineral density [6]. Calcium plays an important role in cellular signaling and physiological functions, from bone mineralization, and cardiac and vascular muscle contraction, to early stages of embryonic neurogenesis, synaptogenesis, neurotransmission and their regulation [7]. In humans, single nucleotide polymorphisms in *ATP2B1* have been implicated as risk factors for essential hypertension; [8] this was underpinned by vascular smooth muscle cell-specific *Atp2b1*-ko mice which showed significantly higher systolic blood pressure with increased intracellular calcium concentration [9]. Rahimi et al. recently reported twelve patients harboring heterozygous *ATP2B1* missense or truncating variants, manifesting mild to moderate global developmental delay. The authors proposed haploinsufficiency as the disease-causing mechanism [10]. Functional Ca^2+^ imaging in cells transfected with *ATP2B1* missense variants showed significantly decreased Ca^2+^ extrusion capacity, providing compelling evidence implicating *ATP2B1* in the pathogenesis of a neurodevelopmental phenotype due to dysregulation of Ca^2+^ homeostasis and signaling [10].

## Materials and methods

### Exome sequencing and analysis

Genomic DNA was extracted from peripheral blood via standard protocols. Whole exome sequencing was performed with Agilent SureSelect Clinical Research Exome V1 (Santa Clara, CA, USA) on NextSeq500 (Illumina, CA, USA) (Supporting Methods).

***Splicing RNA experiments, Western blot and [Ca***^***2+***^***]***_***i***_ ***imaging*** are described in Supporting Methods.

## Results

The male (46,XY) proband was born to healthy, non-consanguineous European parents. Cleft palate, micro-retrognathia, unilateral (left) fixed talipes equinovarus, and intermittent stridor were documented at birth. Growth parameters at birth were normal (p25-75) for weight, length, and occipitofrontal circumference (OFC). CGH-array (Affymetrix CytoScan, hg19) did not detect clinically significant genomic imbalances. Detailed phenotyping in early childhood reveals asymmetrical growth deficiency (OFC p50, weight and height p2-10) and distinctive craniofacial appearance characterized by bitemporal narrowing, short palpebral fissures, periorbital fullness, pinched nose, long philtrum, microstomia, bifid uvula, full-saggy cheeks, and micro-retrognathia (Supplementary Fig. S1A, B). Musculoskeletal manifestation includes pectus carinatum, a shortened left Achilles tendon, bilateral brachydactyly with bulbous fingertips, fifth-finger clinodactyly, and flexion deformity of the distal interphalangeal joint of the third and fourth fingers. He has similar digital appearances of the feet with bilateral fourth and fifth-toe syndactyly (Supplementary Fig. S1C–E). Radiographs of the hand show brachymesophalangy (brachydactyly type-A4), characterized by under-modelled phalanges with irregular metaphyses. The middle phalanges of the fifth and index fingers appear dysplastic, resembling an angel-shape phalanx (Supplementary Fig. S1F, G). He was diagnosed with moderate intellectual disability; WISC-IV-Full Scale Intelligence Quotient score of 50 and significantly impaired adaptive functioning. Brain magnetic resonance imaging (MRI) showed periventricular nodular heterotopia (PVNH) in the left lateral ventricle. There is no record of epilepsy.

In retrospect, subclinical hypocalcemia was recorded in the neonatal period and at ten and fourteen years of age (Table 1). Secondary hyperphosphatemia was evident from school age. He maintains a normal renal function. Intact-Parathyroid hormone (iPTH) levels were in the lower limit of normal despite hypocalcemia. Serum magnesium and urinary calcium and phosphate levels were normal. Electroencephalograms, renal ultrasonography, echocardiogram, and ophthalmological assessments were unremarkable. Autism Diagnostic Observation Schedule 2 (ADOS-2) assessment indicates a low likelihood of autism. Recorded systolic and diastolic blood pressure measurements are within the normal limits for age and height. The proband’s parents are phenotypically normal with no history of neurodevelopmental deficit or hypertension. The mother has low-normal adjusted calcium, and normal phosphate and PTH levels. These are normal in the father (Table 1).

**Table 1:** Serum concentration of calcium metabolism indices in the proband and parents.

Assay	Reference interval	Proband				Mother	Father
		Neonate	6 y	10 y	14 y		
Calcium (adjusted)	2.10–2.55 mmol/L	1.70	2.01	2.05	2.05	2.10–2.20	2.34
Intact-Parathyroid Hormone (iPTH)	3.00–8.50 pmol/L	NA	1.85	3.00	3.00	6.00	4.00

*NA* not available, *y* years.

### Exome data analysis

**Fig. 1:**
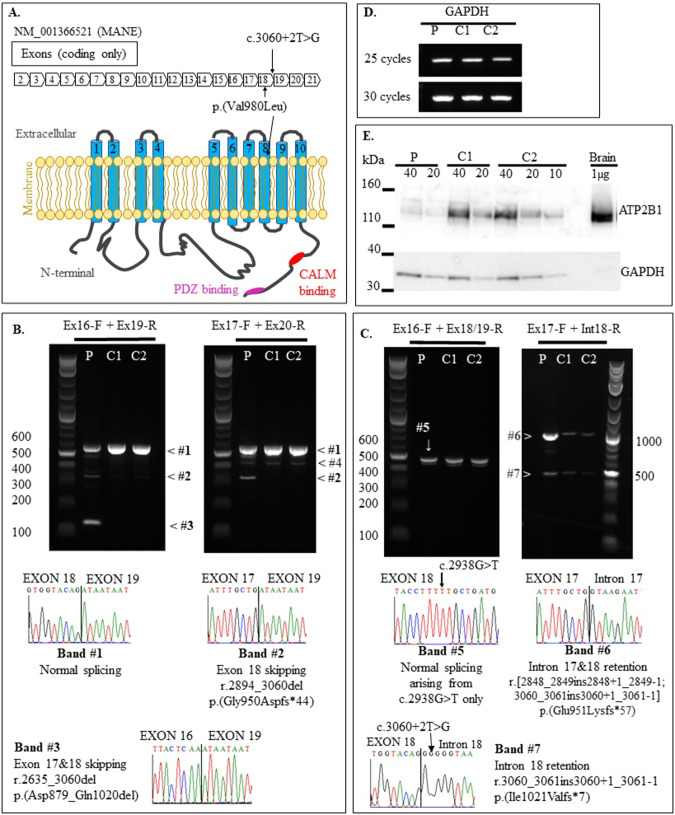
ATP2B1 gene/protein diagram and abnormal splicing events induced by c.3060+2T > G. **A.** Schematic diagram showing location of ATP2B1:c.2938G > T in exon 18 and c.3060+2T > G in intron 18. Ten transmembrane domains, calmodulin (CALM), and PDZ binding domains are depicted in ATP2B1 protein. **B** Splicing effects of ATP2B1 c.3060+2 T > G. RT-PCR amplicons (RNA from proband’s fibroblasts) using two sets of primers (Ex16-F + Ex19-R; Ex17-F + Ex20-R) flanking the c.3060+2 T > G variant (intron 18), we detect two abnormally sized bands in the proband: Band#2 (exon 18 skipping; r.2849_3060del, p.(Gly950Aspfs*44)) and #3 (exon 17–18 skipping; r.2635_3060del, p.(Asp879_Gln1020del)). **C** Using forward primer in exon 16 (Ex16-F) and reverse that flanks exon 18-exon 19 boundary (Ex18/19-R), we detect normally spliced Band#5; Sanger sequencing of proband’s Band#5 established that all normally spliced ATP2B1 transcripts contain c.2983 G > T, p.(Val980Leu) variant only. Using a forward primer in exon 17 (Ex17-F) and reverse in intron 18 (In18-R) we detect increased levels of intron 18 retention (Band#7; r.3060_3061ins3060 + 1_3061-1, p.(Ile1021Valfs*7)) and intron 17&18 retention (Band#6; r.[2848_2849ins2848 + 1_2849-1;3060_3061ins3060 + 1_3061-1], p.(Glu951Lysfs*57)) in proband relative to two controls. All proband’s splice transcripts with intron 18 retention contained splice variant c.3060+2 T > G (Band#7 Sanger sequence) **D** Amplification of GAPDH demonstrates cDNA loading. **E** Western blot of patient and control fibroblasts showing ATP2B1 protein levels. 10–40 μg of fibroblast lysate was loaded. Human brain was used as a positive control. GAPDH was used as a loading control. Image is representative of three independent experiments. Lanes: Proband (P) Control 1 (C1), Control 2 (C2).

Exome variant analysis using de novo, autosomal-dominant (inherited from mosaic parent), X-linked and autosomal-recessive (homozygous) models did not detect any variants relevant to proband’s phenotype. Applying an autosomal-recessive compound heterozygous filter, *ATP2B1* variants were identified: maternally-inherited canonical splice-site variant (GRCh37/NC_000012.11:g.89996818 A > C; NM_001366521.1:c.3060+2 T > G) in intron 18 and paternally-inherited missense (GRCh37/NC_000012.11:g.89996942 C > A; NM_001366521.1:c.2938 G > T,p.(Val980Leu)) in exon 18 which encodes the transmembrane domain 8 (Fig. 1A). Both variants are absent or extremely rare in the population and disease databases (Supplementary Table S3).

### Functional consequences of ATP2B1 variants

**Fig. 2:**
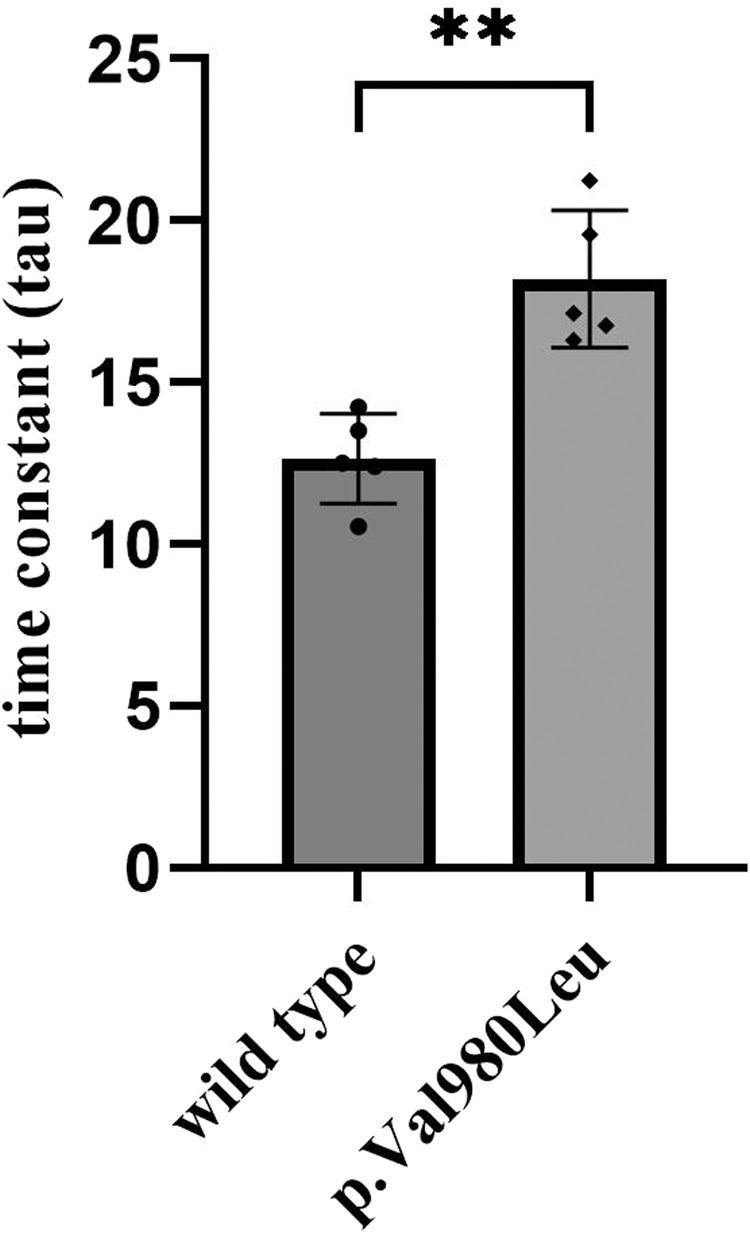
Time-dependent [Ca^2+^]_i_ decline of p.(Val980Leu) transfected HEK293 was analysed after final addition of EGTA that is represented by the time constant tau. Data presented as mean and standard deviation from five independent experiments; **p = 0.0012.

*ATP2B1* cDNA analysis from proband’s fibroblasts demonstrates that the c.3060+2 T > G variant induces multiple abnormal splicing events likely resulting in nonsense-mediated decay (Fig. 1B, C and Supporting Results). Western blot analyses on proband’s fibroblast lysate showed a significant reduction of full-length (135 kDa) ATP2B1 to approximately 20% (Fig. 1D, E and Supporting Results). [Ca^2+^]_i_ imaging experiments revealed p.(Val980Leu) causes decreased cellular Ca^2+^ extrusion compared to the wild type (Fig. 2).

Collective results from our functional studies indicate the mechanism for the disease is due to loss of canonically spliced transcripts from the maternal allele and reduced levels of a dysfunctional ATP2B1 p.(Val980Leu) from the paternal allele.

## Discussion

We describe the first case of biallelic, compound heterozygous *ATP2B1* variants in a proband with a neurodevelopmental phenotype, characterized by distinctive craniofacial and skeletal features, and persistent hypocalcemia due to primary hypoparathyroidism. The Genome Aggregation Database (gnomAD) shows *ATP2B1* is highly intolerant to genetic variation, depleted in both loss-of-function (pLI score of 1) and missense variants (*Z*-score 5.29). Global germline knockout of *Atp2b1* in mice is associated with embryonic lethality [4, 5]. There is a strong association between lethal murine recessive knockouts and genes linked to severe phenotypes in humans, with 75% of known prenatal-lethal genes in humans linked to developmentally lethal phenotypes in mice [11].

Murine studies establish marked surges in *Atp2b1* expression during embryogenesis at the early stage of differentiation into neural progenitor cells, during mitosis and later stages of neural tube formation, postulated to promote neural tube closure in neuronal migration [7, 12]. It is therefore plausible that significant disruption of *ATP2B1* function in human brain predisposes to a neurodevelopmental and malformation phenotype. PVNH is a well-described entity of disordered neuronal migration. The manifestation of partial PVNH in the proband strengthens the hypothesis that *ATP2B1* plays an essential role in the early stages of neurogenesis and PVNH as a phenotypic spectrum of the proposed neurodevelopmental malformation syndrome. Ten out of twelve probands with monoallelic (de novo) *ATP2B1* variants described by Rahimi et al. [10], are reported to have normal brain imaging. Imaging findings described in the remaining two individuals are not indicative of neuronal migration disorders (cerebral cavernoma and isolated ventriculomegaly).

We compare and contrast the phenotypic features of individuals with monoallelic and biallelic pathogenic *ATP2B1* variants. We included the phenotypic data of seven individuals recorded on Decipher, heterozygous for de novo copy number variants; deletions sized 5.3–101 Mb, encompassing *ATP2B1*. Although the phenotypes in individuals with deletions are more likely to represent the effect of the contiguous-gene deletion, we are able to identify and exclude phenotypic features less likely to be attributed to *ATP2B1*. The phenotypic analysis demonstrated some overlap, as well as delineated the distinguishing distinctive features in the monoallelic and biallelic cohorts. We identified the phenotypic features specific for the biallelic loss-of-function effect, which includes PVNH, Pierre-Robin sequence, primary hypoparathyroidism, brachymesophalangy, and a distinctive craniofacial gestalt. (Supplementary Table S5)

While the conspicuous neurodevelopmental deficit in the proband with biallelic *ATP2B1* variants overlaps with the global developmental delay (mild to moderate) observed in the de novo monoallelic cohort [10], the parents of our proband, heterozygous for pathogenic *ATP2B1* variants do not show overt clinical symptoms, including the c.3060+2 T > G variant carrier mother. Pre-mRNA splicing studies indicate c.3060+2 T > G is associated with multiple mis-splicing events, all resulting in probable loss-of-function (encoded premature termination codons or deletion of multiple transmembrane domains). Western blot did not identify any potentially part-functional, truncated ATP2B1 isoforms in fibroblasts from the proband. Three individuals in the cohort described by Rahimi et al., harbor heterozygous truncating *ATP2B1* variants; two with unknown inheritance (c.458 G > A; p.(Trp153*), c.1789C>T; p.(Arg597*)) and one (c.2632 C > T, p.(Gln878*)) confirmed de novo in a patient with motor and speech delay, hyperactivity, normal growth, sparse eyebrow and palpebral edema. Based on the analyses of the NGS and Sanger sequencing data in our study, the proband’s parents were non-mosaic for *ATP2B1* variants (variant allele frequencies were 58%) in the father (c.2938 G > T; p.(Val980Leu)) and 50% in the mother (c.3060+2 T > G). In the absence of mosaicism in the parents, the mechanism behind monoallelic missense and truncating *ATP2B1* variants leading to the phenotypes described remains speculative.

The functional evidence of monoallelic pathogenic *ATP2B1* variants in altering intracellular calcium homeostasis is indisputable. Functional Ca^2+^ imaging data indicates the p.(Val980Leu) variant has a clear deleterious impact on the ATP2B1 function (*tau*-value:18.2; wild-type: 12.64). Rahimi et al. in their publication identified p.(Arg991Gln) and p.(His459Arg) as the high and low-impact variants, respectively. The corresponding *tau*-values were 23.92 and 17.79 [10]. Based on the *tau*-value, p.(Val980Leu) is predicted a low-impact variant. The correlation between *tau*-value and phenotypic severity is not yet delineated. The clinical manifestation in heterozygous carriers of loss-of-function autosomal recessive alleles is likely to be highly variable. The low impact p.(Val980Leu) variant and variable expressivity provide a plausible explanation for the phenotypically unaffected parents harboring heterozygous pathogenic *ATP2B1* variants.

In conclusion, we provide a detailed phenotypic characterization of a biallelic *ATP2B1* disorder, in a proband manifesting a distinctive neurodevelopmental, craniofacial, and skeletal phenotype, and persistent hypocalcemia secondary to primary hypoparathyroidism, discernible from other well-described syndromes (Supplementary Table S2). We provide functional evidence from RNA and protein studies, and functional Ca^2+^ imaging, confirming the deleterious effects of the compound heterozygous *ATP2B1* variants. We postulate a likely critical threshold for retained functional ATP2B1 protein that enables survival and pathogenesis of the clinical phenotype. We have not identified another proband with biallelic *ATP2B1* variants through GeneMatcher [13] and other disease databases. We consider that surviving, manifesting probands with biallelic *ATP2B1* variants may be rare. Detailed phenotyping of more cases with confirmed genotype may delineate and expand the phenotypic and genotypic spectrum of monoallelic and biallelic *ATP2B1*-related disorders, providing insights into the critical role of ATP2B1 in human development.

### Web resources

ClinVar database: https://www.ncbi.nlm.nih.gov/clinvar/ (accessed July 15, 2022)

Genome Aggregation Database (gnomAD): http://gnomad.broadinstitute.org/ (accessed July 15, 2022)

NHLBI Exome Sequencing project (ESP): https://evs.gs.washington.edu/ (accessed July 15, 2022)

Missense3D-DB: http://missense3d.bc.ic.ac.uk/ (accessed July 15, 2022)

Geno2MP: https://geno2mp.gs.washington.edu/Geno2MP (accessed July 15, 2022)

MyGene2: https://www.mygene2.org/MyGene2/ (accessed July 15, 2022)

Decipher: https://www.deciphergenomics.org/ddd/ (accessed July 15, 2022)

Decipher: https://www.deciphergenomics.org/gene/ATP2B1/patient-overlap/cnvs (accessed June 1, 2023)

Global Variome shared LOVD-ATP2B1: https://databases.lovd.nl/shared/variants/ATP2B1 (accessed July 15, 2022)

International Mouse Phenotyping Consortium (IMPC): https://www.mousephenotype.org/data/genes/MGI:104653 (accessed July 15, 2022)

### Supplementary information

Supportive_Information

Figure S1

## Data Availability

The accession numbers for identified *ATP2B1* variants were deposited in ClinVar (https://www.ncbi.nlm.nih.gov/clinvar/) with the following identifiers: SCV002061315 and SCV002061316. We do not have patient consent to release raw next generation sequencing data.
