# Supplementary material for: Biallelic ATP2B1 variants as a likely cause of a novel neurodevelopmental malformation syndrome with primary hypoparathyroidism
Source: Eur J Hum Genet. 2023 Nov 6;32(1):125–9. doi: 10.1038/s41431-023-01484-9 (PMC10772071; doi:10.1038/s41431-023-01484-9)
Supplement: Supplementary file 1 — Supportive_Information [file 41431_2023_1484_MOESM1_ESM.docx]

**SUPPORTING INFORMATION**

# Biallelic ATP2B1 variants as a likely cause of a novel neurodevelopmental malformation syndrome with primary hypoparathyroidism

Patrick Yap^1,2^, Lisa G. Riley^3,4^, Purvi M. Kakadia^1,5^, Stefan K. Bohlander^1,5^, Ben Curran^1^, Meer Jacob Rahimi^6^, Salam Alburaiky^2^, Ian Hayes^2^, Henry Oppermann^6^, Cristin Print^1^, Sandra T. Cooper^4,7,8^*, Polona Le Quesne Stabej^1^*

^1^ Department of Molecular Medicine and Pathology, University of Auckland, Auckland, New Zealand

^2^ Genetic Health Service New Zealand - Northern hub, Auckland, New Zealand

^3^ Rare Diseases Functional Genomics, Kids Research, The Children’s Hospital at Westmead and The Children’s Medical Research Institute, Sydney, NSW 2145, Australia

^4^ Specialty of Child & Adolescent Health, Sydney Medical School, University of Sydney, Sydney, NSW 2006, Australia

^5^ Leukaemia and Blood Cancer Research Unit, Department of Molecular Medicine and Pathology, University of Auckland, Auckland, New Zealand

^6^ Institute of Human Genetics, University of Leipzig Hospitals and Clinics, Leipzig 04103, Germany

^7^ Kids Neuroscience Centre, Kids Research, Children’s Hospital at Westmead, Sydney, NSW 2145, Australia

^8^ The Children’s Medical Research Institute, 214 Hawkesbury Road, Westmead NSW 2145, Australia

*Joint senior authors

Corresponding author: Patrick Yap, Genetic Health Service New Zealand – Northern Hub, Building 30, 2 Park Road, Grafton, Auckland, New Zealand, E-mail: patricky@adhb.govt.nz

**SUPPORTING METHODS**

**Trio whole exome sequencing and variant analysis**

Sequencing reads passing quality filters were aligned to the reference genome build GRGh37/hg19 using Burrows-Wheeler Aligner (BWA) algorithm and for variant calling, we applied GATK base quality score recalibration, indel realignment, duplicate removal, and performed SNP and INDEL discovery and genotyping using standard hard filtering parameters or variant quality score (1). Copy number Variants in proband and parents were called using LUMPY (v0.2.13) and SVTyper (v0.7.0) (2,3).

The variant analysis was performed using Ingenuity Variant Analysis software version 6.0.2 (www.qiagenbioinformatics.com) from Ingenuity Systems. Sequence variants with call quality ≥ 20.0 and read depth ≥ 10.0 were kept. We filtered the remaining variants to exclude variants with a minor allele frequency greater or equal to 0.1% in gnomAD (version v2.1.1), ExAC (version v0.3.1) the 1000 Genomes Project and NHLBI ESP exomes; unless established pathogenic variant. Kept were frameshift, in-frame indel, nonsense, missense, splice site within 10 bases into intron or predicted to disrupt splicing by MaxEntScan. Kept were variants which fitted *de novo*, dominant (inherited from a mosaic parent) and autosomal recessive compound heterozygous or homozygous inheritance model.

**Skin fibroblasts culture and splicing RNA experiments**

To functionally validate the effects of *ATP2B1* c.3060+2T>G on mRNA splicing, RNA from the proband’s cultured skin fibroblasts was reverse-transcribed to complementary-DNA (cDNA). Fibroblasts from proband’s skin biopsy were cultured and RNA was isolated from fibroblasts using the RNeasy mini kit with RNase-free DNase treatment (Qiagen). cDNA was synthesized using SuperScript™ IV first-strand synthesis system (Invitrogen™) from 500 ng of RNA according to kit instructions. Recombinant Taq DNA polymerase (Invitrogen™) was used for PCRs; 95°C 3 min; 35 cycles 95°C 30 s, 58°C 30 s, 72°C 30-60 s depending on amplicon length; 72°C 10 min. RT-PCR products were analysed on a 1% agarose gel followed by Sanger sequencing of purified bands (GeneJET gel extraction kit, Thermo Fisher Scientific). PCR primer sequences used to amplify and Sanger sequence *ATP2B1* fragments are listed in Sup. Table S1.

**Western blot**

Western blot was performed on fibroblast lysate from patient and two male paediatric controls. Fibroblast pellets were lysed in 125 mM Tris pH 6.8, 4% SDS, 20% glycerol with sonication. Samples were run on an 8% Bis-Tris gel in MOPS buffer according to the manufacturers protocol (Life Technologies). Gels were transferred onto PVDF membrane using NuPAGE transfer buffer (Life Technologies) with 5% methanol, 0.1% SDS. Membranes were blocked in 5% skim milk in 0.1% Tween 20/TBS. Blots were probed overnight at 4°C with 1:100 anti-PMCA1 (ATP2B1) antibody (sc-398413, Santa Cruz), and then re-probed with 1:10 000 anti-GAPDH antibody (G9545, SIGMA) as a loading control. Secondary antibodies were 1:10000 anti-mouse HRP (G-21040, Thermo Scientific) or 1:2500 anti-rabbit HRP (NA934, GE Healthcare) for 2 h at RT. SuperSignal West Atto ECL reagent (Thermo Scientific) was used to detect ATP2B1 bands. Images were analysed with ImageStudio Lite (Li-Cor Biosciences).

**[Ca^2+^]_i_ imaging**

HEK293 cells were transfected with an ATP2B1 p.(Val980Leu) expression plasmid and [Ca^2+^]_i_ imaging experiments were performed as previously described by Rahimi *et al*. (4).

**Variant nomenclature**

Variant descriptions are in accordance with HGVS guidelines and recommendations (5). *ATP2B1* annotations are based on the MANE (Matched Annotation from NCBI and EMBL-EBI) transcript NM_001366521.1.

**Statistical Analysis**

Statistical analysis was carried out using SPSS (IBM, Armonk, USA; Version: 24.0.0.2 64-bit). For pairwise comparisons, a t-test was performed. If not stated otherwise, experiments were carried out in 6-tuplicate (independent experiments) and data is presented as mean ± standard deviation. A p-value <0.05 was presumed to be statistically significant. T-test was performed using GraphPad Prism software (version 9.4.0).

**SUPPORTING RESULTS**

**Whole Exome Sequencing**

No clinically significant variants (SNVs, small indels or CNVs) were detected using autosomal recessive (homozygous), *de novo* and autosomal dominant (including mosaic parent) inheritance model filters. Detection of parental mosaicism was limited by depth of coverage. Mean exome coverage for proband, mother and father were 77.15x, 99.22x and 89.55x, respectively.

The proband’s proposed neurodevelopmental malformation phenotype, partially overlaps with other well-described phenotypes, including Feingold syndrome 1 (MIM #164280), HOXD13 limb morphopathies (MIM #186000) (6), periventricular heterotopia (MIM #300049, #608097, #615544, #617201, #618185, #618918), syndromic Pierre-Robin sequence (7), and Carey-Fineman-Ziter syndrome (MIM #254940) (8,9). In-depth phenotype-driven targeted analyses of trio exome data on related genes did not detect any disease-causing variants (Supplementary Table S2).

**Functional consequences of *ATP2B1* variants**

*In silico* analyses concordantly predict both variants to be damaging (Sup. Table S4). Missense 3D-DB, predicts the ATP2B1 p.(Val980Leu) substitution causes a change between buried and exposed state of the target variant residue, resulting in altered protein conformation (10). Mining of RNA-seq data from the GTEx Project (11) identifies *ATP2B1* exon 18 as a canonical exon, present in all *ATP2B1* isoforms across tissues including the brain, skin fibroblasts and blood.

*ATP2B1* cDNA analysis from proband’s fibroblasts demonstrates that the c.3060+2T>G variant induces multiple abnormal splicing events likely resulting in nonsense mediated decay (Supplementary methods) (Fig. 1B-C): 1) exon-18 skipping encodes a premature termination codon (ATP2B1 r.2849_3060del, p.(Gly950Aspfs*44)) predicted to comply with nonsense-mediated decay (NMD). Any mis-spliced transcripts escaping NMD encode a truncated ATP2B1 lacking 270 amino acids (transmembrane domains 8-10, C-terminal calmodulin and PDZ-binding domains; Fig. 1B-Band#2); 2) skipping of exons 17-18, (ATP2B1 r.2635_3060del, p.(Asp879_Gln1020del)), lacking 141 amino acids (transmembrane domains 6-10; Fig. 1B-Band#3); 3) elevated levels of retention of intron 18 or of introns 17 and 18 (ATP2B1 r.3060_3061ins3060+1_3061-1, p.(Ile1021Valfs*7) and r.[2848_2849ins2848+1_2849-1;3060_3061ins3060+1_3061-1], p.(Glu951Lysfs*57); Fig. 1C-Band#7 and #6). Sequencing of cDNA amplicons corresponding to canonical ATP2B1 mRNA splicing shows that the pool of full-length ATP2B1 mRNA (Fig. 1C-Band#5) arises exclusively from the paternal allele with the c.2938G>T variant, encoding p.(Val980Leu).

Western blot analyses on proband’s fibroblast lysate and two male pediatric controls showed significant reduction of full-length (135 kDa) ATP2B1 to approximately 20% in the proband (Fig. 1E), evidently less than 50% (due to loss of mis-spliced transcripts from the c.3060+2T>G allele), suggestive of reduced stability and/or increased turnover of the p.(Val980Leu) ATP2B1. The antibody used recognizes the N-terminus of ATP2B1 and showed no evidence of lower molecular weight products corresponding to C-terminal truncations or the in-frame deletion p.(Asp879_Gln1020del). As c.2986G>T p.(Val980Leu) variant appeared hemizygous by Sanger sequencing of canonically-spliced ATP2B1 transcripts (Fig. 1C/Band #5) in the proband, the full-length ATP2B1 observed on Western blotting (Fig. 1E) is likely comprised only of p.(Val980Leu). Functional Ca2+ imaging shows significantly reduced Ca2+ extrusion capability in HEK293 cells transfected with the p.(Val980Leu) missense variant compared to wild type ATP2B1 (Fig. 2). Collective results from our functional studies indicate the mechanism for disease is due to loss of canonically spliced transcripts from the maternal allele and reduced levels of a dysfunctional ATP2B1 p.(Val980Leu) from the paternal allele.

**SUPPORTING TABLES**

**Sup. Table S1.** Primers used for *ATP2B1* cDNA analysis.

| **Primer Name** | **Sequence (5ʹ - 3ʹ)** | **Expected product size** | **Figure** |
| --- | --- | --- | --- |
| Ex16-F | TGATTGTTGCTTTTACGGGCG | 566 bp | Fig. 2B |
| Ex19-R | GCCCCAGAGTAATGTTCCCA |  |  |
| Ex17-F | GGATACACTCGCTTCCCTGG | 549 bp | Fig. 2B |
| Ex20-R | TGTGTTCCATGACCAGCTTCT |  |  |
| Ex16-F | TGATTGTTGCTTTTACGGGCG | 476 bp | Fig. 2C |
| Ex18/19-R | CTGCACAATTATTATCTGTACCACA |  |  |
| Ex17-F | GGATACACTCGCTTCCCTGG | 948 bp | Fig. 2C |
| In18-R | CCAAGGCAACAGTTCCACAC |  |  |

**Sup. Table S2.** Phenotype-driven targeted analysis based on genes of interest: syndromes with phenotypic overlap the proband’s phenotype.

| **Syndrome/Phenotype** | **MIM #** | **Gene (inheritance)** | **Mean coverage (Proband)^1^** |
| --- | --- | --- | --- |
| Feingold syndrome 1 | 164280 | *MYCN* (AD) | 67.7x |
| Synpolydactyly 1 | 186000 | *HOXD13* (AD, AR) | 104.6x |
| PVNH 1 | 300049 | *FLNA* (XLR) | 48.6x |
| PVNH with microcephaly | 608097 | *ARFGEF2* (AR) | 121.2x |
| PVNH 6 | 615544 | *ERMARD* (AD) | 80.5x |
| PVNH 7 | 617201 | *NEDD4L* (AD) | 115.4x |
| PVNH 8 | 618185 | *ARF1* (AD) | 77.5x |
| PVNH 9 | 618918 | *MAP1B* (AD) | 71.1x |
| Catel-Manzke syndrome | 302380 | *TGDS* (AR) | 59.8x |
| Cerebrocostomandibular syndrome | 117650 | *SNRPB* (AD) | 57.9x |
| TARP syndrome | 311900 | *RBM10* (XLR) | 46.4x |
| Carey-Fineman-Ziter syndrome | 254940 | *MYMK* (AR) | 72.1x |

Legend to Sup. Table 2: PVNH, periventricular nodular heterotopia; AD, autosomal dominant; AR, autosomal recessive; XLR, X-linked recessive, ^1^coding exons (CDS) of MANE select transcripts were used to calculate mean coverage.

**Sup. Table S3.** Presence of *ATP2B1* variants in population and genotype-phenotype databases

| **Database** | **NM_001366521.1**  **c.2938G>T p.(Val980Leu)** | **NM_001366521.1**  **c.3060+2T>G** |
| --- | --- | --- |
| gnomAD2.1.1 | No | No |
| gnomAD3.1.1 | No | No |
| NHLBI ESP | Yes  (1/13,005 Alleles) | No |
| Geno2MP | No | No |
| MyGene2 | No | No |
| LOVD (*ATP2B1*) | No | No |
| Decipher | No | No |
| ClinVar | No | No |

**Sup. Table S4.** *In silico* analysis of *ATP2B1* variants

| **NC_000012.11:g.89996942C>A; NM_001366521.1:c.2938G>T p.(Val980Leu)** | | |  |
| --- | --- | --- | --- |
| ***in silico* prediction tool** | **Prediction** | **Score** | |
| SIFT | Deleterious | 0.01 | |
| PolyPhen | Probably Damaging | 0.987 | |
| CADD phred score | High | 28.1 | |
| FATHMM | Damaging | -4.24 | |
| GERP++ |  | 53.36 | |
| LRT | Deleterious | 0 | |
| MetaLR | Damaging | 0.9686 | |
| MetaSVM | Damaging | 1.0864 | |
| MutationAssessor | High | 4.245 | |
| MutationTaster | Disease-causing | 1 | |
| PROVEAN | Damaging | -2.85 | |
| REVEL |  | 0.902 | |
| Missense 3D-DB* | Damaging |  | |
| **NC_000012.11:g.89996818A>C; NM_001366521.1:c.3060+2T>G** | | |  |
| SSF (Alamut) | Ablation of 5’ splice site; predicts a potential cryptic splice 4 bp downstream of the native intron 18 5’splice | | |
| MaxEnt (Alamut) |  |  |  |
| NNSPLICE (Alamut) |  |  |  |

* Missense 3D-DB, predicts the ATP2B1 p.(Val980Leu) substitution results in a change between buried and exposed state of the target variant residue and is predicted to cause structural damage (10).

**Sup. Table S5.** Phenotypic spectrum of affected individuals with monoallelic and biallelic pathogenic *ATP2B1* variants

| **Phenotypic spectrum** | ***De novo* monoallelic *ATP2B1*^1^ (n)**  **(Total patients = 12)** | ***De novo* contiguous gene deletion encompassing *ATP2B1*^2^ (n)**  **(Total patients = 7)** | **Compound heterozygous biallelic *ATP2B1*^3^**  **(*Proposed distinguishing phenotypic features*)** |
| --- | --- | --- | --- |
| GDD/ID | Mild (6)  Moderate (3)  Unclassified (3) | GDD/ID present in all patients | Moderate ID |
| Structural brain anomaly (imaging) | CCM (1)  Isolated VMG (1) | Corpus callosum dysgenesis and VMG (1)* | ***PVNH*** (left lateral ventricle) – no epilepsy |
| Craniofacial gestalt | Non-specific, no recognisable gestalt | Non-specific, no recognisable gestalt | ***Short palpebral fissures, pinched nose, Pierre-Robin sequence, bifid uvula, saggy cheeks, microstomia*** |
| Congenital cardiac anomaly | Septal defect (1)  TGA (1)  AoR dilatation (1) | PS and VSD (1) | None |
| Musculoskeletal anomaly | Marfanoid habitus (2)  Short stature (2)  Pectus deformities (2)  Scoliosis (2) | Shoulder-girdle abnormality, lumbar lordosis (1)  Growth deficiency (1)* | Postnatal-onset asymmetrical growth deficiency  Pectus deformity  Talipes equinovarus |
| Digital anomaly | Clinodactyly (2)  Arachnodactyly (2) | Abnormal thumb (1) | ***Brachydactyly type A4***  ***4/5-toe syndactyly***  ***Flexion deformity of the interphalangeal joints*** |
| Others | NA | Microtia (1)  Anterior chamber ocular anomaly (1)**  Pigmentary skin anomaly (2) | ***Primary hypoparathyroidism*** – persistent hypocalcemia and low-normal PTH |
| Comments |  | *PPP1R12A* and *DUSP5* are associated with a proposed AD HI phenotypes |  |

**Key**: **^1^**Patients described in Rahimi *et al* (4); **^2^**Patients submitted on from Decipher Genomics (Decipher:<https://www.deciphergenomics.org/gene/ATP2B1/patient-overlap/cnvs>); **^3^**Proband described in this paper; ( n): number of affected patients in the cohort; **GDD:** global developmental delay; **ID:** intellectual disability; **CCM:** cerebral cavernous malformation; **VMG**: ventriculomegaly; **PVNH**: Periventricular nodular heterotopia; **TGA**, transposition of great arteries; **PS:** pulmonary stenosis; **VSD:** ventricular septal defect; **AoR:** aortic root; **PTH**: parathyroid hormone; **NA**: not available; *****17Mb deletion including *PPP1R12A;* ******5.2Mb deletion including *DUSP6*; **AD:** autosomal dominant; **HI:** haploinsufficiency.

**SUPPORTING FIGURE**


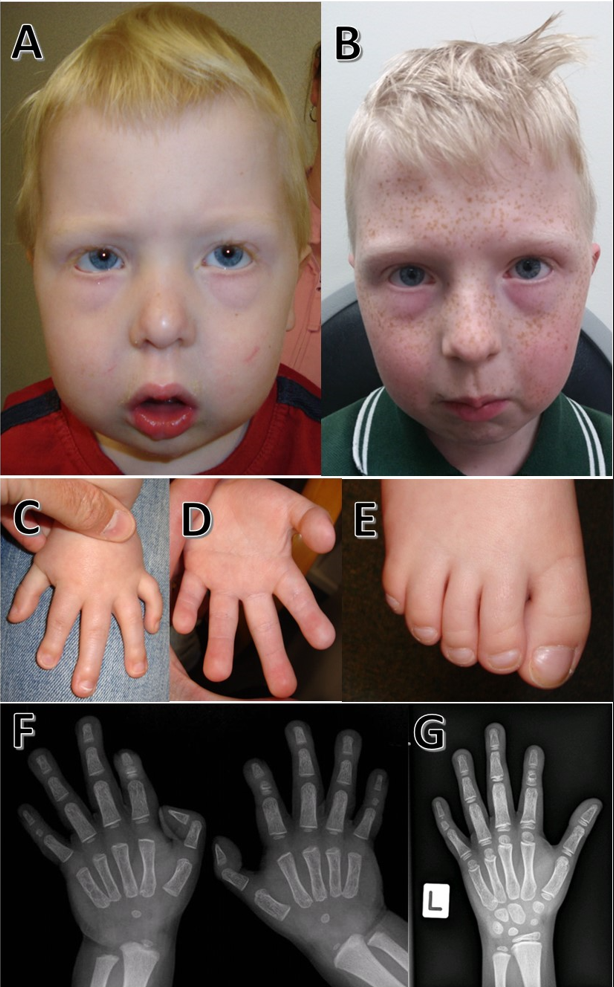


**Legend to Sup. Figure S1. A** Craniofacial gestalt at 2y4m. **B** Craniofacial gestalt at 10y2m. **C,D,E**: Hand and foot configuration, brachydactyly with bulbous fingertips, cutaneous syndactyly of the 4th and 5th toes. **F-G** Radiographs of the hands at 2y4m and 10y2m. Dysplastic middle phalanges of index and fifth fingers, under-modelled phalanges, angel-shape phalanx of the middle phalanx of the index finger. Key: y-years; m-months.

**SUPPORTING REFERENCES**

1. Poplin R, Ruano-Rubio V, DePristo MA, Fennell TJ, Carneiro MO, Auwera GA Van der, et al. Scaling accurate genetic variant discovery to tens of thousands of samples. bioRxiv. 2017 Jul 24;201178.

2. Layer RM, Chiang C, Quinlan AR, Hall IM. LUMPY: A probabilistic framework for structural variant discovery. Genome Biol. 2014 Jun 26;15(6):R84.

3. Chiang C, Layer RM, Faust GG, Lindberg MR, Rose DB, Garrison EP, et al. SpeedSeq: ultra-fast personal genome analysis and interpretation. Nat Methods. 2015 Oct;12(10):966–8.

4. Rahimi MJ, Urban N, Wegler M, Sticht H, Schaefer M, Popp B, et al. De novo variants in ATP2B1 lead to neurodevelopmental delay. Am J Hum Genet. 2022 May;109(5):944–52.

5. den Dunnen JT, Dalgleish R, Maglott DR, Hart RK, Greenblatt MS, Mcgowan-Jordan J, et al. HGVS Recommendations for the Description of Sequence Variants: 2016 Update. Hum Mutat. 2016 Jun 1;37(6):564–9.

6. Temtamy SA, Aglan MS. Brachydactyly. Orphanet J Rare Dis. 2008;3(1):15.

7. Tan TY, Farlie PG. Rare syndromes of the head and face—Pierre Robin sequence. WIREs Dev Biol. 2013 May 1;2(3):369–77.

8. Alrohaif H, Töpf A, Evangelista T, Lek M, McArthur D, Lochmüller H. Whole-exome sequencing identifies mutations in &lt;em&gt;MYMK&lt;/em&gt; in a mild form of Carey-Fineman-Ziter syndrome. Neurol Genet. 2018 Apr 1;4(2):e226.

9. Di Gioia SA, Connors S, Matsunami N, Cannavino J, Rose MF, Gilette NM, et al. A defect in myoblast fusion underlies Carey-Fineman-Ziter syndrome. Nat Commun. 2017;8(1):16077.

10. Khanna T, Hanna G, Sternberg MJE, David A. Missense3D-DB web catalogue: an atom-based analysis and repository of 4M human protein-coding genetic variants. Hum Genet. 2021 May 1;140(5):805–12.

11. Lonsdale J, Thomas J, Salvatore M, Phillips R, Lo E, Shad S, et al. The Genotype-Tissue Expression (GTEx) project. Nat Genet. 2013;45(6):580–5.
