# Supplementary figures and images for: Biallelic ATP2B1 variants as a likely cause of a novel neurodevelopmental malformation syndrome with primary hypoparathyroidism
Source: Eur J Hum Genet. 2023 Nov 6;32(1):125–9. doi: 10.1038/s41431-023-01484-9 (PMC10772071; doi:10.1038/s41431-023-01484-9)

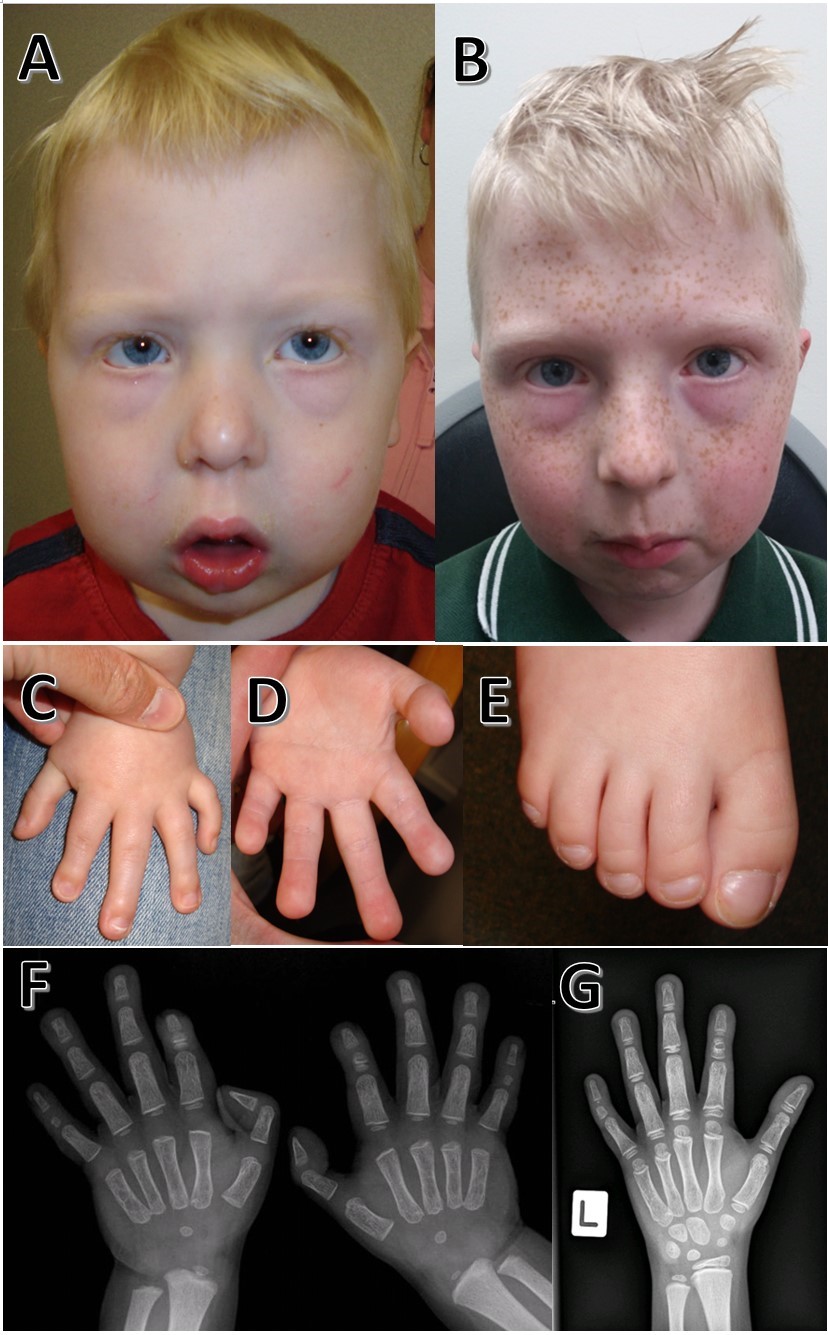

Supplement: Supplementary file 2 — Figure S1 [file 41431_2023_1484_MOESM2_ESM.jpg]
